# Spatiotemporal target selection for intracranial neural decoding of abstract and concrete semantics

**DOI:** 10.1093/cercor/bhac034

**Published:** 2022-02-15

**Authors:** Keisuke Nagata, Naoto Kunii, Seijiro Shimada, Shigeta Fujitani, Megumi Takasago, Nobuhito Saito

**Affiliations:** Department of Neurosurgery, The University of Tokyo, 7-3-1 Hongo, Bunkyo-ku, Tokyo 113-8655, Japan; Department of Neurosurgery, The University of Tokyo, 7-3-1 Hongo, Bunkyo-ku, Tokyo 113-8655, Japan; Department of Neurosurgery, The University of Tokyo, 7-3-1 Hongo, Bunkyo-ku, Tokyo 113-8655, Japan; Department of Neurosurgery, The University of Tokyo, 7-3-1 Hongo, Bunkyo-ku, Tokyo 113-8655, Japan; Department of Neurosurgery, The University of Tokyo, 7-3-1 Hongo, Bunkyo-ku, Tokyo 113-8655, Japan; Department of Neurosurgery, The University of Tokyo, 7-3-1 Hongo, Bunkyo-ku, Tokyo 113-8655, Japan

**Keywords:** abstract and concrete, brain–machine interface, electrocorticography, language, semantic decoding

## Abstract

Decoding the inner representation of a word meaning from human cortical activity is a substantial challenge in the development of speech brain–machine interfaces (BMIs). The semantic aspect of speech is a novel target of speech decoding that may enable versatile communication platforms for individuals with impaired speech ability; however, there is a paucity of electrocorticography studies in this field. We decoded the semantic representation of a word from single-trial cortical activity during an imageability-based property identification task that required participants to discriminate between the abstract and concrete words. Using high gamma activity in the language-dominant hemisphere, a support vector machine classifier could discriminate the 2-word categories with significantly high accuracy (73.1 ± 7.5%). Activities in specific time components from two brain regions were identified as significant predictors of abstract and concrete dichotomy. Classification using these feature components revealed that comparable prediction accuracy could be obtained based on a spatiotemporally targeted decoding approach. Our study demonstrated that mental representations of abstract and concrete word processing could be decoded from cortical high gamma activities, and the coverage of implanted electrodes and time window of analysis could be successfully minimized. Our findings lay the foundation for the future development of semantic-based speech BMIs.

## Introduction

In recent years, there has been growing interest in the acquisition and translation of brain signals into behavioral outputs that compensate for impaired brain function. In particular, unraveling linguistic information using intracranial signals has emerged as a topical area in the field of neural decoding, as it provides the possibility of speech neuroprosthesis for individuals with locked-in syndrome and severe anarthria (Birbaumer 2006; Guenther et al. 2009; Brumberg and Guenther 2010; Bocquelet et al. 2016). Brain mapping and task-oriented neurocognitive studies have revealed functional models of speech processing, thereby providing clues to neural speech decoding. Based on extant research on speech processing pathways, 3 major representations, i.e. semantic, auditory, and articulatory, have been proposed as potential targets for neural speech decoding (Rabbani et al. 2019). Semantic aspects of speech or the meaning of words are first retrieved as lexical concepts, which are subsequently processed into an acoustic form of lemma and phonological codes. Finally, articulation is performed via syllabification and phonetic encoding (Indefrey 2011). As semantic processing is the most upstream process of the interpretation of speech representation, it is considered the most intuitive and versatile platform for speech brain–machine interfaces (BMIs) (Wang et al. 2011; Rabbani et al. 2019).

Although semantic aspect of speech is expected to be a novel target of speech decoding, progress in neurocognitive research concerning semantic processing has been limited due to difficulties in defining the properties of speech semantics. In contrast to auditory and articulatory decoding, in which the final output is evaluated using a relatively simple quantitative parameter such as the acoustic spectrum or kinetics of speech and articulatory organs, how to quantify speech semantics has been a major issue. According to the attribute-based view, a concept that defines an object can be decomposed into a set of features or semantic elements and encoded into semantic information according to the probability, weight, and importance of each attribute (Rosch 1978). For example, the word “bird” can show high accordance with the semantic attributes such as “animal,” “having wings,” and “flying” although it shows less or no probability for a semantic feature such as “having 4 legs.” More superordinate factors such as abstractness and concreteness are also evaluated as one of the attributes which describes a specific word. The semantic aspects of a word can be quantified by considering these semantic attributes and their probability weights as vectors and plotting them in a semantic feature space.

Previous neurocognitive studies have revealed that semantic attributes in various hierarchies can be extracted from cortical activity using blood-oxygen-level-dependent (BOLD) imaging in functional magnetic resonance imaging (MRI) (Binder et al. 2005; Wang et al. 2013), magnetoencephalography (Travis et al. 2013), functional near-infrared spectroscopy (Zinszer et al. 2018), and electrocorticography (ECoG) (Wang et al. 2011; Rupp et al. 2017). An ECoG study by Rupp et al. (2017) provided direct evidence supporting attribute-based encoding models and demonstrated that each semantic attribute was further encoded to ECoG signals as specific spectral, spatial, and temporal patterns via conceptual retrieval.

With regard to individual attributes representing speech semantics, word abstractness is one of elemental features that constitutes word meaning. The mechanism by which brain discriminates between abstract and concrete concepts has been of key interest in cognitive neuroscience, as it provides insight into the neurocognitive frameworks underlying conceptual knowledge processing. In semantic recognition, dual-coding theory is a widely supported theory that proposes that linguistic and nonlinguistic (or imagery-based) systems work in parallel in abstract and concrete word processing (Paivio 1971, 1986). The linguistic system is responsible for coding semantic information of both abstract and concrete concepts, while nonlinguistic systems associated with sensorimotor imagination are involved in the retrieval of concrete concepts. Paivio (1986) proposed that a verbal-based system is lateralized in the language-dominant hemisphere and a nonverbal system is distributed over the bilateral hemispheres. This theory is supported by neurocognitive evidence indicating that abstract concepts activate a strongly left-lateralized network, while concrete concepts evoke bilateral multimodal and association cortices (Binder et al. 2005).

Functional MRI studies have provided insight into the neural bases of abstract and concrete word processing (Binder et al. 2005; Indefrey 2011; Wang et al. 2011, 2013). However, the application of functional MRI to practical BMIs has been limited due to the lack of sufficient temporal resolution for decoding ongoing streams of continuous speech and the need for large signal-recording equipment that is unsuitable for daily use (Bocquelet et al. 2016). These disadvantages may be overcome by employing ECoG as a signal platform due to its high spatial and temporal resolution and compact signal-processing devices. Powers in the high gamma frequency range are robust spectral targets of ECoG neural decoding and correlate with event-related cortical activation (Crone et al. 1998) associated with increased firing rates of neural cell populations below the recording electrode (Buzsáki et al. 2012). High gamma powers (HGPs) also exhibit temporal and spatial correlations with functional MRI BOLD responses during the processing of semantic information of abstract and concrete word status (Kunii et al. 2013). Previous ECoG studies using subdural electrodes have reported discrimination among multiple object categories in a picture-naming task (Wang et al. 2011; Matsuo et al. 2015). Nevertheless, there is a paucity of ECoG-based evidence for the neural decoding of specific semantic elements of words. Regarding this, confirming the results of previous functional MRI studies and focusing on more specific temporal and spatial targets of neural decoding using ECoG may be the first step toward semantic-based speech BMIs.

The present study aimed to evaluate the dynamics of semantic word processing and to identify the spatiotemporal targets of abstract and concrete semantic dichotomy from the perspective of neural decoding. We conducted semantic decoding using ECoG high gamma activity with a language task to evaluate the tactile imageability of the displayed word based on its abstractness and concreteness.

## Materials and methods

### Participants

Study participants included patients over 16 years of age with intractable epilepsy who underwent subdural electrode implantation for clinical purposes. Among 39 patients who underwent subdural electrode implantation at the University of Tokyo between January 2016 and December 2020, we selected 14 hemispheres from 14 patients with more than 100 electrodes implanted in the left or right hemisphere, covering both the inferior frontal gyrus (IFG) and superior temporal gyrus (STG). None of the participants had more than 100 electrodes implanted in both hemispheres. Participants were evaluated using the Wechsler Adult Intelligence Scale (3rd or 4th edition, depending on the case) before electrode implantation. Two patients with a full-scale intelligence quotient (FIQ) less than 65 were excluded from further analysis.

The 12 participants who fulfilled the inclusion criteria performed a language task and underwent ECoG recordings during the task. Eight participants underwent electrode implantation in the left hemisphere, while 4 participants underwent electrode implantation in the right hemisphere. Language dominance was evaluated using functional MRI before electrode implantation and subsequently confirmed with bed-side cortical electric stimulation via the implanted electrodes using a method described previously (Kunii et al. 2011). These assessments revealed that the language-dominant side was on the left in all participants with the exception of one whose electrodes were implanted in the right hemisphere. Thus, data were recorded from the dominant hemisphere in 9 participants and from the nondominant hemisphere in 3 participants.

The location of the implanted electrodes was determined solely based on the clinical purpose, depending on each patient’s suspected epileptic foci and in consideration of adjacent eloquent cortices. We selected electrodes placed on the lateral aspect of each hemisphere and the postbasal region of the temporal lobe as the target of analysis. The participants’ characteristics, profiles of the implanted electrodes, and results of neuropsychological assessment including FIQ and verbal comprehension index (VCI) are shown in Table 1. Electrode locations of each participant are illustrated in Supplementary Figure S1. Written informed consent was obtained from all participants prior to enrollment. The study protocols complied with the principles of Declaration of Helsinki and the study was approved by the Institutional Review Board of the University of Tokyo (approval number 1797).

**Table 1:** Participant characteristics.

Participant no.	Age (years)	Implanted hemisphere	Language dominancy	Channel numbers	FIQ	VCI	Epileptic foci
P1	56	Lt	Lt	188	92	104	Lt temporal lobe
P2	26	Rt	Rt	220	108	109	Rt middle frontal lobe
P3	17	Lt	Lt	220	122	133	Rt occipital lobe (ganglioglioma)
P4	39	Lt	Lt	188	95	88	Lt temporal lobe (cortical dysplasia)
P5	44	Lt	Lt	192	66	73	Lt temporal lobe (hippocampal sclerosis)
P6	51	Lt	Lt	188	72	66	Multiple (post encephalitis)
P7	22	Lt	Lt	228	85	86	Lt temporal lobe
P8	19	Lt	Lt	240	74	80	Lt temporal lobe
P9	33	Lt	Lt	188	105	96	Lt temporal lobe
P10	57	Rt	Lt	128	80	92	Bilateral temporal lobe (Rt > Lt)
P11	26	Rt	Lt	156	69	79	Bilateral temporal lobe (Rt > Lt)
P12	32	Rt	Lt	188	68	71	Rt frontal + temporal lobe

*Notes*: FIQ = full-scale intelligence quotient; Lt = left; Rt = right; VCI = verbal comprehension index.

### Semantic task paradigm

**Fig. 1:**
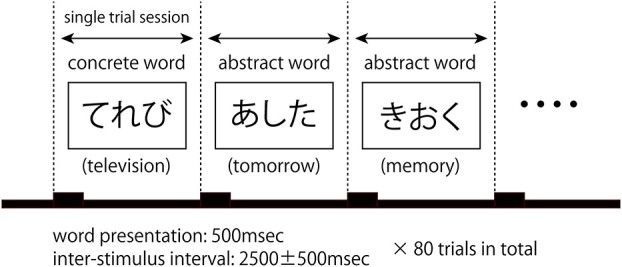
Semantic task paradigm. A single trial was composed of a word presentation period (500 ms) and interstimulus interval (2500 ± 500 ms). Japanese Hiragana words in “abstract” or “concrete” semantic categories were presented in a pseudorandomized order 80 times. Participants were instructed to create a mental image about the tangibleness of what the presented word referred to. ECoG was recorded throughout the task paradigm.

A property identification task focusing on the tactile imageability of the displayed word was performed as follows: Participants were seated in an electrically shielded room and instructed to look at a computer monitor that presented visual stimuli. The word set prepared for this task consisted of 20 abstract and 20 concrete words which were chosen from the Word List by Semantic Principles, provided by Center for Corpus Development, National Institute for Japanese Language and Linguistics (https://github.com/masayu-a/WLSP-familiarity), so that word familiarity scores were matched for both word sets. Word sets and their familiarity scores are presented in Supplementary Table SS1. In each trial, a stimulus was displayed as a Japanese Hiragana word (consisting of 3 letters or 3 syllables) on a computer monitor. Participants were instructed to decide whether the concept represented by the displayed word was tangible (concrete word, e.g. “television”) or intangible (abstract word, e.g. “tomorrow”). Participants were instructed to create a mental image of the tangibleness of the displayed word but not to verbalize the answer. Twenty words in each word category were presented twice in a pseudorandomized order, yielding 80 stimuli in total. Word presentation lasted for 500 ms each, and the intertrial interval between stimuli was 2500 ± 500 ms. ECoG signals during the task were recorded and were used to decode the displayed word category using machine learning. The task paradigm is illustrated in Fig. 1.

### Data acquisition and signal processing

ECoG signals were digitally recorded at a sampling rate of 2000 Hz. Reference electrodes were placed in the subdural space that was furthest from the assumed epileptic foci, with the recording surface of the electrodes facing the dural side. ECoG data analyses were performed using custom software written in MATLAB R2021a (MathWorks, Natick, MA, USA). All waveforms were recalculated using an average reference. Trials involving excessive epileptic activity were identified visually and removed from further analyses.

We selected high gamma activity as the signal platform for this decoding study. To extract HGP, ECoG signals were band-pass filtered between 70 and 150 Hz, and the Hilbert transform was applied. Data from each channel were cut in each trial from 500 ms pre- to 2000 ms post-stimulus presentation. The HGP in each trial was further divided into time bins of 250 ms, and the average HGP for each bin was calculated. Averaged bin power from 0 ms to 1500 ms (6 time bins) was used in the present analysis.

### Classification

A linear support vector machine (SVM) classifier was used to decode semantic categories (abstract or concrete) from single-trial cortical activity recorded as ECoG HGP. The dataset consisted of 80 trials, which were divided into training and test sessions data (training 79 trials, test one trial). HGP from −500 to 0 ms in all trials within the training session was used as the baseline. All trial data, including training and test sessions, were baseline-normalized and converted to Z scores. The SVM classifier was constructed using the normalized training data and then tested against the normalized test data of same participants. Cross-validation was performed using a leave-one-out scheme, which provided 80-fold validation.

Prior to training the SVM, we performed a feature selection procedure to select feature dimensions that contributed to the classification of the 2 groups. The procedure was performed for each validation fold. Each single-trial session consisted of a 2-dimensional feature matrix (channel and time bin). For each time bin in each channel, Wilcoxon’s rank sum test was performed to compare Z scores of bin powers obtained in abstract and concrete word presentation within the training data. Feature dimensions with *P*-values greater than 0.05 were excluded from the further classification step. Based on the retained feature dimensions, the corresponding Z scores of the bin powers were selected as the final feature vectors to train the SVM classifier. Data from each test (containing one trial) were then inputted to the trained classifier, which finally outputted a predicted label of the displayed word category. Decoding accuracy was calculated as the proportion of correct output labels in the test session over 80 folds.

### Analysis of contributing brain regions

To identify brain regions and time bins with the greatest contribution to the classification, we evaluated the temporal and spatial distributions of the retained feature dimensions which were used to train the SVM. For each feature dimension (channel and time bin), we calculated the averaged HGP (normalized using a baseline of −500 to 0 ms) of all 40 trials corresponding to abstract and concrete word presentations. The deviation index (DI) was used to evaluate quantitative differences in cortical reactivity to abstract and concrete word status. The DI was calculated as follows:

(1) \begin{eqnarray*} &&\mathrm{Deviation}\ \mathrm{index}\ \left(\mathrm{DI}\right)\nonumber\\ &&=\left(\mathrm{averaged}\ \mathrm{HGP}\_\mathrm{abstract}-\mathrm{averaged}\ \mathrm{HGP}\_\mathrm{concrete}\right)/\nonumber\\ &&\left(\mathrm{averaged}\ \mathrm{HGP}\_\mathrm{abstract}+\mathrm{averaged}\ \mathrm{HGP}\_\mathrm{concrete}\right)\nonumber\\\end{eqnarray*}

A positive DI indicated dominance in abstract word processing, whereas a negative DI indicated dominance in concrete word processing. Feature dimensions that contributed to classification were also evaluated using the predictor weights of the SVM, derived for each feature from the linear regression model of the trained classifier.

To quantify the contribution of each brain region, we assigned each electrode to the superior/middle/inferior frontal gyrus, superior/middle/inferior temporal gyrus, angular gyrus, supramarginal gyrus, and primary sensorimotor cortex (SM1). This assignment procedure was performed by visual inspection of the 3-dimensional model of electrodes on each participant’s brain, which was constructed as previously described (Ishishita et al. 2019) using the postimplantation computed tomography (CT) data and preoperative T1-weighted image data. To visualize the across-individual results, preoperative MRI and coordinated postoperative CT were both normalized using Montreal Neurological Institute coordinates via linear scale adjustment using SPM12 (The Wellcome Centre for Human Neuroimaging, UCL Queen Square Institute of Neurology, London, UK). The coordinates for each electrode were extracted and overlaid on the surface rendering of a normalized brain using FreeSurfer ver. 6.0.0 (https://surfer. nmr.mgh.harvard.edu/). The DIs and predictor weights were averaged across participants within each brain region. The DIs and weights of the features that were excluded in the feature selection procedure were assumed as zero.

## Results

### Prediction accuracy

**Fig. 2:**
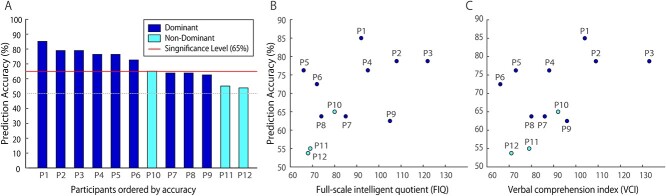
(a) Prediction accuracy of each participant. Significance level (65%) and chance level (50%) are shown as red and gray lines, respectively. Of 9 participants whose data were recorded from the dominant hemisphere, 6 gained prediction accuracy over the significance level, while 3 participants with electrodes in the nondominant hemisphere did not exceed the significance level. Average accuracy of the dominant hemisphere (73.1 ± 7.5%) was significantly higher than that of the nondominant hemisphere (57.9 ± 6.2%) (*P* = 0.0225, Welch’s *t*-test). (b) Relationship between prediction accuracy and full-scale intelligent quotient. A statistically significant correlation was not observed (*r* = 0.505, *P* = 0.094). (c) No statistically significant correlation was observed between prediction accuracy and VCI (*r* = 0.518, *P* = 0.091).

The prediction accuracy of each participant is shown in Fig. 2. The significance level of prediction accuracy was calculated as 65% based on the binomial distribution of 80 trials (*P* < 0.05). Six out of 9 participants exceeded the significance level in the dominant hemisphere, resulting in a total accuracy of 73.1 ± 7.5%. Three subjects with electrodes in the nondominant hemisphere exhibited a prediction accuracy of 57.9 ± 6.2%. Decoding results in the dominant hemisphere were significantly higher than those in the nondominant hemisphere (*P* = 0.0225, Welch’s *t*-test, two-tailed). Dominant hemispheres tended to have more channels than nondominant hemispheres, but there was no significant correlation between number of channels and prediction accuracy (*r* = 0.245, *P* = 0.442, Pearson’s correlation coefficient). We did not observe any significant correlation between prediction accuracy and age of the participants (*r* = 0.205, *P* = 0.521), FIQ (*r* = 0.505, *P* = 0.094), and VCI (*r* = 0.518, *P* = 0.091).

### Overview of high gamma activity in each gyrus

**Fig. 3:**
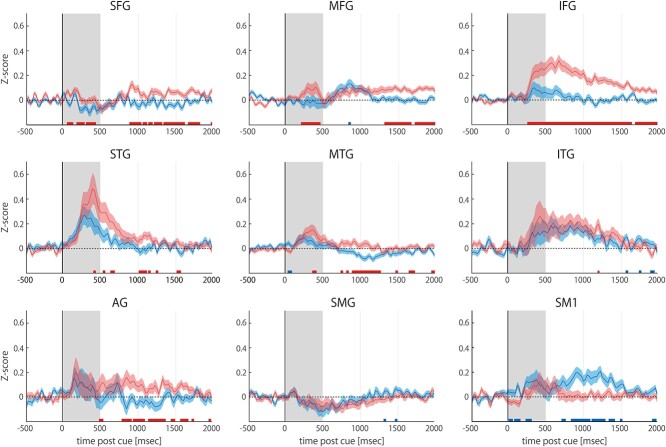
Task-related transition of average HGP across participants. Trial-and-channel averages of HGPs in the language-dominant hemisphere with abstract and concrete word presentations are illustrated as red and blue lines, respectively, with standard error of the mean represented by the shaded areas. The word presentation period (0–500 ms) is represented by the gray shaded background. Significant differences in HGPs between the 2 word categories are represented as red (“abstract” is dominant) and blue (“concrete” is dominant) error bars (*P* < 0.05, Wilcoxon’s rank sum test). The IFG demonstrated prominent activation during abstract word processing from 250 to 1250 ms, while the primary sensorimotor cortex (SM1) presented a delayed peak after 500 ms only during concrete word presentation. Activation in the STG was observed for both word categories, with no significant difference between the categories. SFG = superior frontal gyrus; MFG = middle frontal gyrus; MTG = middle temporal gyrus; ITG = inferior temporal gyrus; SMG = supramarginal gyrus; AG = angular gyrus.

Task-related dynamics of HGPs in each brain region were visualized per gyrus (Fig. 3) by obtaining a trial-and-channel average of HGPs across 9 participants who underwent electrode implantation in their language-dominant hemisphere. HGPs in each gyrus were window-averaged in bin width of 25 ms to evaluate gentle transition of the waveform. Significant differences between HGPs related to abstract and concrete word processing were observed for each time bin using Wilcoxon’s rank sum test (all *P* < 0.05).

The reaction to word presentation peaked from 250 to 500 ms in the STG and middle temporal gyrus. However, no significant difference was observed in peak activity between abstract and concrete word processing. A notable difference was observed in the IFG, in which the average HGP corresponding to abstract word presentation peaked later (from 500 to 750 ms) compared to that in other gyri and the activation persisted even after 1500 ms. Activity associated with abstract word processing demonstrated a significant dominance over that associated with concrete word processing after 250 ms, and the difference was greatest in the delayed time after 500 ms. No significant difference was observed in SM1 between the two categories in the early phase (from 0 to 500 ms), but delayed activation was observed for concrete word processing with a gentle peak around 1000 ms, while abstract words did not activate the gyrus after 500 ms. The middle frontal gyrus (MFG) demonstrated mild activation of HGP from 500 to 1000 ms during concrete word presentation, but no significant difference between the two categories was observed.

### Contributing brain regions and temporal targets of machine learning

**Fig. 4:**
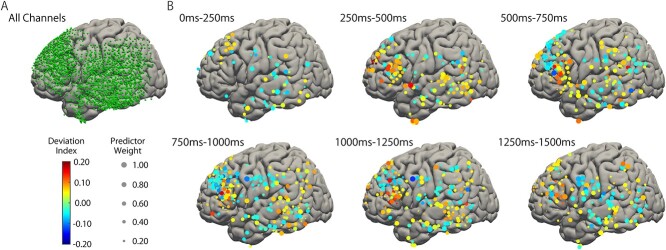
Spatiotemporal transition of cortical activation corresponding to abstract and concrete word processing. (a) All channels adopted in our research are plotted on a standard brain as green dots. (b) Spatial and temporal distribution of DIs and predictor weights in each channel and time bin, plotted on a standard brain. Channels that were excluded in the feature selection procedure are not shown in this figure. Warm and cool colored dots illustrate positive and negative DIs, respectively, while the size of the dots represents predictor weights.

**Fig. 5:**
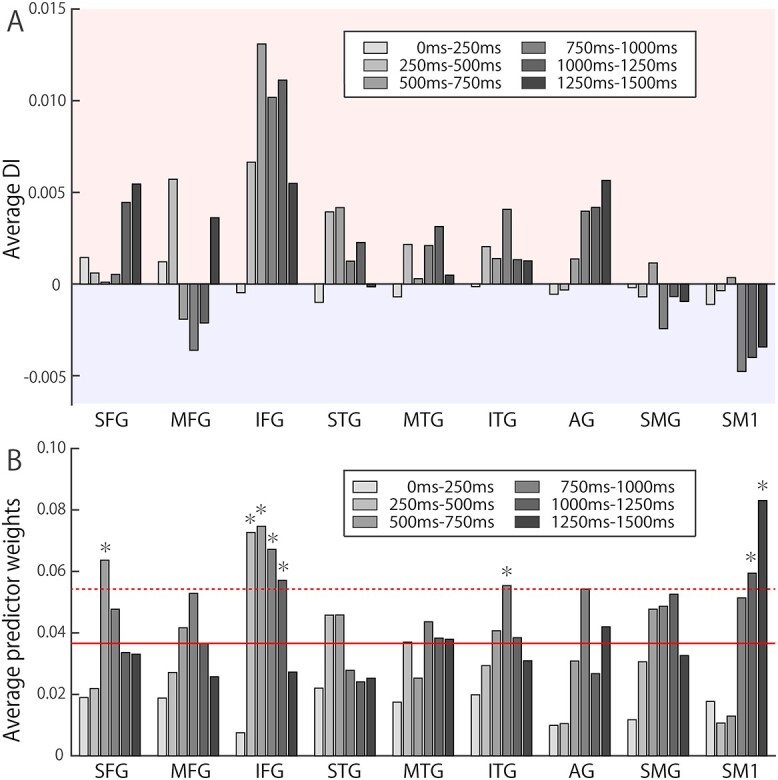
Functional dynamics of each brain region. DIs and predictor weights of each feature dimension in the language-dominant hemisphere were averaged across participants within each brain region. (a) Spatiotemporal dynamics of averaged DIs are presented. A positive DI average (dominant for abstract words) was predominantly observed in the IFG, whereas a negative DI average (dominant for concrete words) was observed in later time components in the primary sensorimotor cortex (SM1). (b) Spatial and temporal distribution of significant predictors are illustrated as averaged predictor weights. Red dotted line represents the average weight of all areas (shown as a red solid line) plus standard deviation; values above this were regarded as significant predictors. Predominantly high predictor weights were observed from 250 to 1250 ms in the IFG and after 1000 ms in the SM1. SFG = superior frontal gyrus; MFG = middle frontal gyrus; STG = superior temporal gyrus; MTG = middle temporal gyrus; ITG = inferior temporal gyrus; AG = angular gyrus; SMG = supramarginal gyrus.

The distribution of electrodes in the dominant hemisphere in nine participants was overlaid on a normalized brain (Fig. 4a). The spatial distributions of the contributing channels in the retained feature dimensions are shown as dot maps for each time bin in Fig. 4b, wherein the colors correspond to DIs and the size represents predictor weights. Channels in the IFG exhibited dominance in abstract word processing in time bins after 250 ms, while the SM1 exhibited dominance in concrete word presentation in later time bins after 750 ms. The MFG was strongly activated from 500 to 1000 ms after concrete word presentation. The response to abstract word presentation in the IFG was notable, with a peak DI from 500 to 750 ms and delayed sustained activity lasting until 1500 ms. These dynamics are quantified in Fig. 5a, which presents the across-participant average of DIs for each gyrus and time bin. A positive DI average (dominant in the abstract category) was predominantly observed in the IFG after 250 ms, while a negative DI average (dominant in the concrete category) was prominent in the SM1 after 750 ms and from 500 to 1250 ms in the MFG.

The temporal and spatial dynamics of the averaged predictor weights are presented in Fig. 5b. Specific regions were considered significant contributors if the classification weights in the gyri were higher than the average weight of all areas plus a standard deviation (Tian et al. 2011; Wang et al. 2019). The average weights were significantly higher in the IFG from 250 to 1250 ms and in the SM1 from 1000 to 1500 ms. These results implied that abstract word discrimination was predominantly influenced by the IFG, while concrete words were characterized by activation of the SM1 in the delayed time phase after 1000 ms.

Given the uneven distribution of the significance predictors, targeted neural decoding with the limited time bins (from 250 to 1500 ms in the IFG and after 1000 ms in the SM1) was performed as described above, which revealed a prediction accuracy of 68.2 ± 8.0% (Table 2). This result was minor but statistically comparable to the result derived from all gyri from 0 to 1500 ms (73.1 ± 7.5%, *P* = 0.284, paired *t*-test, two-tailed). As individual results, the prediction accuracy of participants 1, 2, 3, 5, and 6 decreased in the targeted decoding scheme, while the accuracy of participants 4, 7, 8, and 9 improved using the same method. Reduction of the accuracy of the two decoding schemes (full-feature scheme minus targeted scheme) are also presented in Table 2. We observed no correlation between the reduction of prediction accuracy and numbers of the retained features in IFG (*r* = −0.526, *P* = 0.146) and SM1 (*r* = 0.295, *P* = 0.441), although there was significant correlation between reduction of the accuracy and numbers of the retained features in the brain regions other than IFG and SM1 (*r* = 0.694, *P* = 0.038). Participants whose features were localized in IFG and SM1 tended to show improvement in the targeted decoding schemes, while participants whose features were widely distributed over multiple brain regions (notable in participant 1) were not suitable for reducing analysis targets.

**Table 2:** Decoding results from full features and targeted features.

Participant no.	Prediction accuracy (Full-feature scheme) (%)	Prediction accuracy (Targeted scheme) (%)	Reduction of prediction accuracy (%)	IFG features	SM1 features	The other features	All features
P1	85.0	57.5	27.5	9	22	142	173
P2	78.8	75.0	3.8	21	4	89	114
P3	78.8	60.0	18.8	14	2	60	76
P4	76.3	83.8	-7.5	21	13	33	67
P5	76.3	71.3	5.0	23	4	72	99
P6	72.5	58.8	13.8	23	4	72	99
P7	63.8	71.3	-7.5	17	8	75	100
P8	63.8	67.5	-3.8	29	5	51	85
P9	62.5	68.8	-6.3	16	6	61	83
Average	73.1	68.2	4.9	19.2	7.6	72.8	99.6
SD	7.5	8.0	12.0	5.6	5.9	28.7	29.3

*Notes*: Full-feature scheme (all brain regions, 0–1500 ms); Targeted scheme (inferior frontal gyrus from 250 to 1250 ms and primary sensorimotor cortex from 1000 to 1500 ms); IFG = inferior frontal gyrus; SM1 = primary sensorimotor cortex.

## Discussion

This study aimed to decode the inner mental representation during semantic processing of abstract and concrete words focusing on tactile imageability. Our task did not require specific word output; rather, it represented semantic concept retrieval and haptic imageability-based decision-making. Our classifier could discriminate two semantic representations with high accuracy from high gamma activity in ECoG of language-dominant hemispheres. The decoding results indicated that the dominant hemisphere was more informative than the nondominant hemisphere, especially the IFG and SM1 for abstract and concrete word status, respectively. According to predictor weights derived from the trained classifier, activities in the IFG from 250 to 1250 ms and that in the SM1 from 1000 to 1500 ms were identified as the most significant and spatiotemporally localized targets of neural decoding of abstract and concrete word representations, respectively, which enabled the derivation of comparable decoding results. These findings indicate that significant predictors of haptic imageability during abstract and concrete word processing were clustered in spatial and temporal feature dimensions.

### Spatial localization of abstract and concrete semantics

Functional MRI studies have suggested that semantic aspects of speech activate distributed networks across multimodal areas including frontal, temporal, and parietal association cortices (Pulvermuller et al. 2009; Binder and Desai 2011), posterior cingulate gyrus (Gao et al. 2019), and SM1 (Ekstrand et al. 2017; Dreyer and Pulvermuller 2018). ECoG represents the ubiquitous distribution of semantic information across broad areas of the cerebral cortex, implying an attribute-based encoding model in which semantic word properties are represented as temporal, spatial, and spectral patterns (Rupp et al. 2017). A previous functional MRI study by Wang et al. (2013) examined semantic decoding associated with abstract and concrete word dichotomy. The study reported neural decoding of abstractness and concreteness of words in a covert synonym identification task. The predictive brain regions for abstract words were predominantly located in linguistic systems in the language-dominant hemisphere, whereas predictors for concrete words were distributed over the parietal and temporo-occipital association cortices and MFG for concrete words, which is in support of dual-coding theory.

Our research confirmed that ECoG high gamma activity in the IFG and SM1 provided predictive value for abstract and concrete semantic representations. The present task paradigm focused on the tactile imageability of a single word as a key element of abstract and concrete semantics. Imageability refers to the difficulty with which a word evokes mental imagery (Paivio et al. 1968). Based on the premise that highly concrete words represent high image-arousing value (Paivio et al. 1968), high-imagery and concrete words, and low-imagery and abstract words are often used interchangeably (Ogawa and Nittono 2019). Higher values of imageability are associated with sensorimotor information (Schwanenflugel et al. 1988), supported by neurocognitive evidence that modality-preferential sensorimotor systems are involved in semantic processing of concrete words. In this regard, words concerning visual, auditory, olfactory, and gustatory states specifically activate the corresponding perceptual systems (Kiefer et al. 2008; Barrós-Loscertales et al. 2012), and haptic information processing is strongly associated with sensorimotor cortex (Gao et al. 2019). Further, it has been reported that motor systems are involved in the comparison of semantic similarities in conjunction with sensory systems (Carota et al. 2017), and the sensorimotor cortex contributes to abstract word processing, with activation of the face motor area during nonimagery mental word presentation in the very early stage of semantic processing (Dreyer and Pulvermuller 2018).

The IFG has been reported to play a key role in the differentiation of abstract and concrete semantics (Binder et al. 2005; Della Rosa et al. 2018). One explanation for the function of the IFG is attributed to the verbal semantic network proposed in dual-coding theory (Wang et al. 2011). Della Rosa et al. (2018) argued that abstract and concrete semantics were defined in 2 axes: imageability and context availability. Abstract and concrete words are generally thought to be recognized in association with a network of relevant semantic knowledge or contexts (Schwanenflugel et al. 1988). Low imageability and low context availability have been correspondingly observed for abstract words, evoking the left IFG as a functional convergence zone to integrate the 2 representations (Della Rosa et al. 2018). Indeed, extant research has proposed that the IFG may be a specific target for machine learning in semantic decoding (Bocquelet et al. 2016).

### Temporal targets of neural decoding

In accordance with previous studies, imageability-related brain regions, including the SM1 and IFG, were strongly activated in the present haptic imageability identification task. It is notable that the later-phase activation was prominent in classification, whereas the early component provided minimal contribution to the machine learning results. Our findings indicated that the STG was strongly activated in the early phase after stimulus presentation and the IFG was subsequently activated from 200 to 300 ms with long-lasting activity sustained even after 1500 ms. The SM1 demonstrated a biphasic reaction with early and delayed peak activation. The first activation in the STG did not contribute to the decoding process, whereas delayed, sustained activity in consequently activated regions played key roles in distinguishing semantic categories.

This could be explained by the nature of our semantic task, which consists of 2 distinct steps: the early exogenous phase of semantic recognition of the presented word, and the later endogenous phase of semantic decision-making. Several studies focusing on word recognition reported that the early cortical activation peaking at 400 ms reflected lexico-semantic modulation (Travis et al. 2013) or the process of semantic integration with the existing context (Rugg et al. 1994). Meanwhile, delayed activation of the prefrontal cortex emerging between 500 and 800 ms has been reported to be involved in information searching systems in working memory when evaluating specific characteristics of presented items (West and Holcomb 2000) and represents differences in the imageability of word stimuli (Gullick et al. 2013). The spatial distribution of the activated working memory network for the duration of mental processing encompasses the dorsolateral prefrontal cortex (Ramsey et al. 2006) to left IFG, the latter of which is responsible for the phonological loop (Binder et al. 2005). Our study indicated that the later-phase of the semantic decision-making process, which was partially related to working memory, was more prominent than the early recognition phase as predictors of abstractness and concreteness. This result is consistent with a previous study which reported that verbal working memory is also involved in the discrimination of abstract and concrete semantics (Binder et al. 2005) and serves as a predictor of inner mental representations (Ramsey et al. 2006).

### Implications for future development of semantic neural decoding

Decoding inner speech presentation is necessary for the successful implementation of speech BMIs, whose potential users predominantly comprise individuals with impaired ability of expressing speech, such as patients with locked-in syndrome, amyotrophic lateral sclerosis, and those who underwent laryngectomy (Bocquelet et al. 2016). The semantic processing of a word constitutes the most upstream portion of inner speech, which is untethered to the subsequent step of speech production including auditory and articulatory processing (Rabbani et al. 2019). The concept of semantic-based speech BMIs was first proposed by Wang et al. (2011) as a natural, intuitive, and fast communicating interface for individuals with impaired speech ability and even for patients who have injury in brain regions concerning auditory and articulatory processing of speech. However, its feasibility remains unclear from the perspective of implementation to practical speech BMI.

This study demonstrated that the inner decision-making process of a specific semantic attribute could be extracted from ECoG high gamma activity. Although this result is not practical enough to be directly applied to speech BMI by itself, there is a possibility that a combination of multiple semantic attributes could illustrate inner mental representation of the framework of a word meaning, as presented in the attribute-based encoding model. Semantic decoding is also expected to be utilized in combination with auditory and articulatory decoding (Rabbani et al. 2019), and possibly serve as self-monitoring or self-modifying option together with the existent decoding scheme.

The problem of invasiveness should also be considered when envisioning future realization of safe and practical BMIs, especially in ECoG platforms in which implantation of large electrodes tends to be required. We presented here that predictors of abstractness and concreteness were clustered in spatial and temporal feature dimensions. This result highlighted the possibility of minimizing the spatiotemporal targets of machine learning with minimum information loss, but individual differences in the distribution of contributing brain regions should be taken in account. Further research is required to confirm whether targeted decoding schemes could be generalized to the other semantic attributes.

### Study limitations

Although the present task design enabled us to evaluate semantic decision-making process based on mental imagery without vocalization, the covert nature of the task required careful interpretation of the task results. It is difficult to determine whether the participant is actively participating in the task, and we did not record participants’ comments or reactions to the task to confirm that the participants’ mental imagery was identical to what we intended as the task purpose. Mental imagery of “abstract” and “concrete” could be varied for each individual participant, given that semantic processing of abstract and concrete words is strongly related to individual experiences, social environments, and emotions (Dreyer and Pulvermuller 2018; Mkrtychian et al. 2019). Thus, our study only focused on the partial and personal aspects of a broad framework of concepts represented by abstract and concrete semantics. Individual cortical activity also varied for each participant, reflected in the difference in reactivity to the targeted decoding scheme. Individual difference in how widely the retained features were distributed should be considered when planning to apply this targeted decoding scheme. To interpret the individual difference of cortical reactivity to the semantic task, an evaluation of each participant’s behavioral outputs as a post-task comment or interview should be included in the task design.

In ECoG studies, it was also a limitation that the spatial coverage of electrodes was restricted to the areas of clinical interest (Crone et al. 2006). Since the electrodes were placed around the peri-Sylvian traditional language area in most participants, we were unable to perform whole-brain analysis similar to that performed in functional MRI. This precluded investigation of the parietal and occipitotemporal association cortices and posterior cingulate gyrus, which are reported to associated with concrete word processing. Another limitation is that the study was conducted at a single center with a small cohort; therefore, participants’ demographic variability is limited, and the study results rely upon the expertise of the researchers involved.

## Conclusion

Based on haptic imageability, mental representations of abstract and concrete word processing could be decoded from cortical high gamma activities. Although semantic representations are distributed over a wide cortical network, each region and time component have specific weights for neural decoding, indicating that coverage of the implanted electrodes and time range analysis may be minimized depending on the required accuracy. We believe that these findings will eventually pave the way to the future development of semantic-based speech BMIs.

## Authors’ contributions

Conceptualization: N.K., K.N.; Methodology: N.K., S.S., K.N.; Investigation: S.S., M.T., S.F.; Formal Analysis: K.N.; Writing – Original Draft: K.N.; Writing – Review & Editing: N.K.; Funding Acquisition: N.K.; Supervision: N.S.

## Funding

This work was supported in part by a Grants-in-Aid (No. 19 K09452) for Scientific Research (C) from the Japan Society for the Promotion of Science.

## Conflict of interest statement

The authors declare no competing interests.

## Data availability

The datasets presented in this article are not readily available because the participants of this study did not agree for their data to be publicly shared. Requests to access the datasets should be directed to the corresponding author. Formal data sharing agreement is required to provide the datasets. Scripts used for the analyses are available at GitHub (https://github.com/kskngt/sematic-decoding).

## Supplementary Material

Supplementary_material_bhac034
