# Supplementary material for: Spatiotemporal target selection for intracranial neural decoding of abstract and concrete semantics
Source: Cereb Cortex. 2022 Feb 15;32(24):5544–54. doi: 10.1093/cercor/bhac034 (PMC9753048; doi:10.1093/cercor/bhac034)
Supplement: Supplementary_material_bhac034 [file supplementary_material_bhac034.pdf]

**Abstract word: Japanese**	**Abstract word: English translation**	**Familiarity score**	**Concrete word: Japanese**	**Concrete word: English translation**	**Familiarity score**
Ashita	Tomorrow	2.13	Amado	Shutter	1.87
Gaman	Patience	1.76	Botan	Button	1.86
Fusei	Injustice	1.93	Denchi	Battery	1.93
Jikan	Time	2.19	Hagaki	Postcard	1.98
Kakugo	Readiness	1.82	Hechima	Loofah	1.74
Kehai	Sign	1.92	Katana	Sword	2.02
Kinen	Anniversary	1.92	Kusuri	Drug	2.06
Kioku	Memory	2.05	Makura	Pillow	2.04
Kisetsu	Season	1.66	Naifu	Knife	1.80
Kitai	Expectation	1.87	Nimotsu	Baggage	2.10
Konya	Tonight	2.04	Saifu	Wallet	2.08
Kotoba	Language	1.91	Tabako	Cigarette	1.75
Masatsu	Friction	1.72	Taiko	Drum	2.02
Mirai	Future	1.75	Tansu	Wardrobe	2.26
Riyuu	Reason	2.06	Tatami	Tatami mat	2.02
Saigo	Last	1.81	Terebi	Television	2.02
Seigi	Justice	1.55	Tokei	Clock	1.82
Shinpo	Progress	1.93	Tsukue	Desk	2.11
Chikara	Power	2.12	Yakan	Kettle	1.87
Zengo	Back and front	1.83	Zukan	Picture book	2.01

Supplementary Table 1 Word list used for the language task

A word list of Japanese abstract and concrete words used for the current language task and their English translations are described above. 20 words for each category were chosen from the Word List by Semantic Principle (WLSP), released online by Center for Corpus Development, National Institute for Japanese Language and Linguistics (https://github.com/masayu-a/WLSP-familiarity). Word familiarity scores were also listed in the WLSP as subordinate evaluation. Familiarity scores in average were 1.90 ± 0.16 in abstract words, while 1.97 ± 0.13 in concrete words. There was statistically no difference in familiarity of two-word categories (P = 0.1561, Welch's t-test, two-tailed).

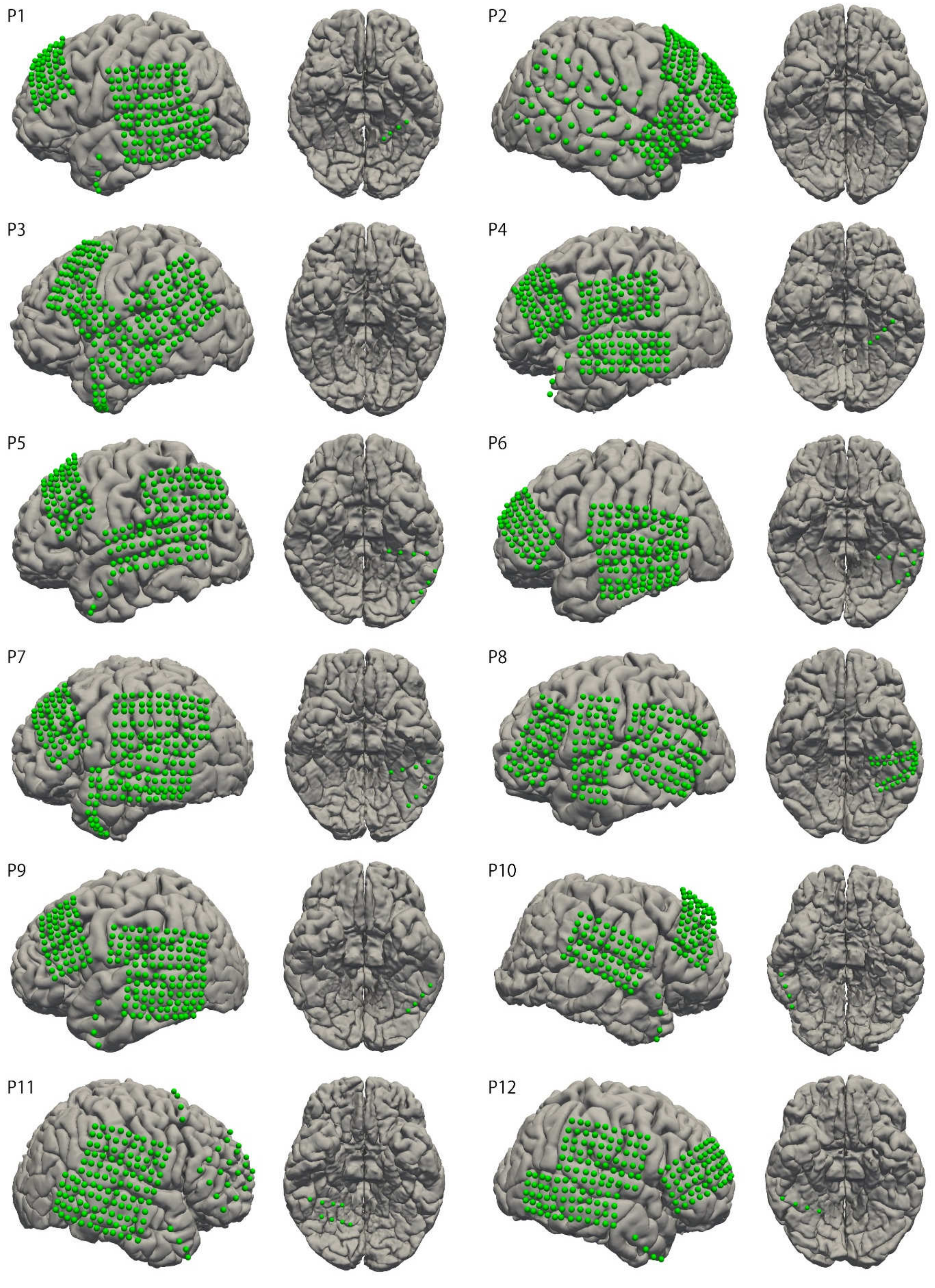

Supplementary Fig. 1 Electrode location of each participant. The electrodes placed on the lateral aspect of each hemisphere and the postbasal region of the temporal lobe was selected as the target of analysis. Electrodes used for the present study are displayed as green dots on the 3-dimension model of participants' normalized brains.
